# Critical Epitopes in the Nucleocapsid Protein of SFTS Virus Recognized by a Panel of SFTS Patients Derived Human Monoclonal Antibodies

**DOI:** 10.1371/journal.pone.0038291

**Published:** 2012-06-12

**Authors:** Li Yu, Li Zhang, Lina Sun, Jing Lu, Wei Wu, Chuan Li, Quanfu Zhang, Fushun Zhang, Cong Jin, Xianjun Wang, Zhenqiang Bi, Dexin Li, Mifang Liang

**Affiliations:** 1 Laboratory Institute for Viral Disease Control and Prevention, China CDC, Beijing, China; 2 Shandong Key Laboratory for Infectious Disease Prevention and Control, Shandong Province CDC, Jinan Shandong, China; Technical University of Braunschweig, Germany

## Abstract

**Background:**

SFTS virus (SFTSV) is a newly discovered pathogen to cause severe fever with thrombocytopenia syndrome (SFTS) in human. Successful control of SFTSV epidemic requires better understanding of the antigen target in humoral immune responses to the new bunyavirus infection.

**Methodology/Principal Findings:**

We have generated a combinatorial Fab antibody phage library from two SFTS patients recovered from SFTSV infection. To date, 94 unique human antibodies have been generated and characterized from over 1200 Fab antibody clones obtained by screening the library with SFTS purified virions. All those monoclonal antibodies (MAbs) recognized the nucleocapsid (N) protein of SFTSV while none of them were reactive to the viral glycoproteins Gn or Gc. Furthermore, over screening 1000 mouse monoclonal antibody clones derived from SFTSV virions immunization, 462 clones reacted with N protein, while only 16 clones were reactive to glycoprotein. Furthermore, epitope mapping of SFTSV N protein was performed through molecular simulation, site mutation and competitive ELISA, and we found that at least 4 distinct antigenic epitopes within N protein were recognized by those human and mouse MAbs, in particular mutation of Glu10 to Ala10 abolished or significantly reduced the binding activity of nearly most SFTS patients derived MAbs.

**Conclusions/Significance:**

The large number of human recombinant MAbs derived from SFTS patients recognized the viral N protein indicated the important role of the N protein in humoral responses to SFTSV infection, and the critical epitopes we defined in this study provided molecular basis for detection and diagnosis of SFTSV infection.

## Introduction

Severe fever with thrombocytopenia syndrome (SFTS), with average case fatality rate of 12%, is an emerging infectious disease caused by a newly discovered virus, named SFTS virus (SFTSV) [1]. Although the disease has been proved to have viremia in acute phase and the specific antibody responses were appeared in both acute and convalescent phases of SFTS [1], [2], however, the functions of viral structural proteins in immune responses and immune pathogenesis still remain unclear.

SFTSV was classified in the genus *Phlebovirus*, family *Bunyaviridae*, a family of spherical enveloped virus with a tripartite RNA genome of negative or ambisense polarity [3]. Like other known *Bunyavirus*, SFTSV contains three structural proteins, RNA-dependent RNA polymerase (L), glycoproteins (Gn and Gc), and nucleocapsid(N)proteins. The N protein of 245 amino acids, as in the case of many other nucleoproteins of negative strand RNA virus, acts as a scaffold for packing of virus. Antibodies against SFTSV N protein were commonly detected early after infection, which suggested the N protein is possible to be used as target antigen for early diagnosis of SFTSV infection. It has been reported that the viral N protein is highly immunogenic for many members in the genus of *Phlebovirus* and acts as major antigen [4], [5]. Therefore, major epitopes of the SFTSV N antigen and related functional significance need more investigation.

In this report, we for the first time described the generation and characterization of a panel of human monoclonal antibodies (MAbs) against SFTSV N protein using phage display library approach, and the targeted antigenic epitopes were further mapped by competitive assay, molecular modeling and site-directed mutations. The results provided in this study could facilitate understanding of humoral responses to SFTSV infection and help to develop diagnostic tools for detection and diagnosis of SFTSV infection at various infection stages, which could be ultimately applied in clinical work as well as epidemic surveillance.

## Materials and Methods

### Cells, Virus and Purified Virions

The origin and preparation of SFTSV have been described earlier [1]. Briefly, Vero-E6 cells (ATCC CRL-1586) were infected with SFTSV strain HB29 [1] at m.o.i. of 1 and cultivated for 14 days. The medium supernatant containing virus particles of 1.0×10^8^/ml was harvested and removed of cell debris by centrifugation, and further purified using ultracentrifugation with 20% sucrose density gradient. The purified virions were analyzed by SDS-PAGE and electron microscope analysis to confirm the quality of virus particles, and further used as antigen for phage library screening or antibody analysis.

### Construction and Screening of Human Antibody Phage Display Library

The procedures of phage display library in the vector pComb 3H followed the methods described previously [6], [7], [8]. Briefly, lymphocytes were isolated from the blood samples of two SFTS patients in convalescent phase, from Shandong province of China. Total cellular mRNA was extracted using RNeasy Mini kit (Qiagen, Valencia, CA) and cDNA was synthesized with primer oligo (dT) using Transcriptor High Fidelity cDNA Synthesis kit (Roche, Mannheim, Germany). PCR amplification was then performed using FastStrat High Fidelity PCR System (Roche, Mannheim, Germany). The light and heavy chain genes were amplified from the cDNA by PCR using the primer pairs from 5 VK, 7 V L and, 8 VH gene family [9]. The light chain genes were first cloned into the vector pComb 3H with enzymes *Xba* I and *Sac* I, therefore the heavy chain genes were cloned in to the light chain library pool with enzymes *Xho* I and *Spe* I as standard protocol published previously [8], [9]. The initial diversity of the library was evaluated and assured by sequencing of randomly picked over 200 clones for each step of library construction and the complexity of library was then calculated. The final yielded antibody libraries were panned and screened with purified SFTSV virions following the standard panning procedure [10].

### Generation of Human IgG Antibody or Mouse Monoclonal Antibody

After three rounds of phage panning, individual clones from enriched phage pools were analyzed by ELISA against SFTS virions or purified N protein. The positive human Fab clones were sequenced and aligned with the DNAPLOT program for alignment to the VBASE database. The human Fab antibodies selected based on binding activity and sequence variance were converted to human IgG by cloning the Fab genes into baculovirus expression cassette vectors, expressed in SF9 cells, and finally purified with protein A column (GE Healthcare, Sweden) for further characterization and functional analysis. Rabbit polyclonal and mouse monoclonal antibodies against N protein were produced commercially by AbMax Biotechnology company (China). The protocols for animal tests were reviewed and approved by animal care committee of China CDC.

### Production and Purification of Recombinant N Protein

The N protein gene from S segment of SFTSV strain HB29 with His tag was cloned into vector pET30a (Novagen, Madison, USA) and expressed in *Escherichia coli* as we described previously [11]. Briefly, the *E.coli* bacteria (Rosetta2 DE3) transfected with N expression vector plasmid was induced in the presence of 1 mM isopropyl-1-D-thiogalactopyranoside (IPTG), followed by growth at 30°C for 16 h. The protein was obtained from inclusion bodies and purified on an AKTA prime plus system equipped with HisTrap HP column (GE Healthcare, Sweden) for affinity chromatography, and the N protein was eluted at a gradient from 100 to 300 mM imidazole and finally dialyzed with PBS buffer (pH 7. 2). The purity of the protein was confirmed by SDS-PAGE and western blot analysis using SFTS patient sera. The purified N protein then further used for epitope mapping in the study.

### Immunofluorescence Assay (IFA)

IFA was performed on different cells according to the experimental design. Vero-E6 cells infected with SFTSV were used to measure the reactivity of MAbs for viral antigens. The SFTSV N protein or glycoprotein (Gn and Gc) genes were separately cloned into baculovirus vector pAc-UW51 and expressed with recombinant baculovirus/SF9 (ATCC CRL-1711) cells system, the expression of N protein or glycoprotein was confirmed by SFTS patient sera. The N protein or Glycoprotein expressing SF9 cells were used to determine the viral protein binding specificity of human or mouse monoclonal antibodies gained in this study. To measure the binding activity of site-directed mutations, 293T cells were grown to 80%–90% confluence and transfected with pCDNA-N plasmid, using the FuGene reagent (Roche). The cells were washed and fixed on glass slides with acetone. Human or mouse MAbs were incubated for 30 min at 37°C, and bound antibodies were detected by FITC-conjugated goat anti-human or anti-mouse antibody (Sigma), and visualized under immunofluorescence microscope.

### Western Blot Analysis (WB)

The viral proteins from purified SFTS virions were separated by SDS-PAGE under reducing conditions, and transferred to a PVDF membrane. After blocking with PBST (0.01 M PBS with 0.1% Tween-20, pH 7.4) containing 1% BSA for 1 h at room temperature, the membrane-immobilized proteins were probed by purified human MAbs (20 µg/ml) or 1∶50 diluted mouse MAbs from hybridoma ascites overnight at 4°C. Monoclonal goat anti-human or anti-mouse antibody (Sigma) was used as secondary antibody. The bands were visualized by 3, 3′ diaminobenzidine (DAB) according to the manufacturer’s instructions.

### Microneutralization Assay

The microneutralization assay was performed as previously described [1]. Briefly, 2-fold serial dilutions of the 1∶10 diluted human MAbs (1 mg/ml) or mouse MAbs from hybridoma ascites were incubated with a suspension of 100 TCID_50_ of SFTSV strain HB29 for 1.5 hours. The mixture was then incubated with Vero cells in quadruplicate in a 96-well plate for 12 days. Viral infection was detected using immunofluorescence assays with rabbit polyclonal antibody against N protein. The end-point titer was expressed as the reciprocal of the highest dilution factor that prevented infection.

### Competitive ELISA Assay

A panel of extensive competition experiments was developed to evaluate the binding specificity of MAbs and determine whether these MAbs bind to distinct sites in N protein. Purified SFTSV N protein was used as coating antigen in 0.05 M bicarbonate buffer (pH 9.6) and incubated overnight at 4°C. After blocking the plate with PBST containing 1% BSA for 1 h at 37°C, human or mouse MAbs were dissolved in PBS and added into each well by 50 µl in continuous series 2-fold dilution to prepare a concentration gradient of competitors. Then 50 µl HRP-MAbs was then added into each well at a previously decided concentration known to give 90% of the maximum absorbance value on N antigen. After incubation, 3, 3′, 3, 5′ -tetramethylbenzidine (TMB) was added, and absorbance was measured at 450 nm. Percent of binding inhibition of labeled antibodies was calculated according to the formula:



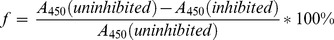



### Molecular Modeling and Docking of the Antibody to N Protein

The computational simulation was carried out using Discovery studio 2.0 (Accelrys, San Diego, CA) [12], [13], [14]. Suitable template was obtained through a BLAST search of the Protein Databank (PDB). The homology modeling of human antibody and SFTSV N protein were performed individually using DS Homology Modeling protocol, and the 3D model of antibody was optimized using Antibody loop refinement protocol. The N protein and antibody were further refined by Energy Minimization and Molecular Dynamic Simulation program under CHARMm forcefield, which provides powerful mechanics for studying the energetic and motion of molecules. The models were validated by Ramachandran plots. Protein-protein docking of N protein with human monoclonal antibody was performed using the ZDOCK and RDOCK program by specifying the antibody residues of the variable region on the binding interface. RDOCK refinement was performed on the top 100 poses of the filtered ZDOCK output, and applied scoring function to each docked structure for best binding models. By visualizing and analyzing the 3D computational interaction, several potential interfaces of the N protein were chosen in further study.

### Amino Acid Substitutions by Site-directed Mutagenesis

A set of site-specific mutations were generated using QuickChange II site-directed mutagenesis kit (stratagene, La Jolla, CA) for PCR using manufacturer’s guidelines. The truncation mutations of N protein were generated from the N-terminal or C-terminal end by every 5 amino acids. All mutations were generated using pCDNA5.0 plasmid as a template. All constructs were transfected to 293T cells for transient expression, and the mutations were analyzed by IFA assay described above for binding activity with human and mouse MAbs.

### Ethical Consideration

According to the medical research regulation of Ministry of Health, all studies involved in human samples were reviewed and approved by the ethics committee of China CDC, which uses international guidelines to ensure confidentiality, anonymity, and informed consent. The written informed consent was agreed by the patients. The protocols for animal tests have been approved by animal care committee of China CDC.

## Results

### Generation and Characterization of Human Antibodies Against SFTSV

Using the pComb 3H vector system, the SFTSV patients derived light chain library was first constructed with a complexity of >1.0×10^7^ clones, the Fd heavy chain genes were efficiently inserted into the light library vectors and finally resulted in a initial combinatorial Fab library with a complexity of >1.0×10^8^ independent clones and 100% Fab genes diversity after sequencing confirmation. After three rounds of phage panning against purified SFTS virions, we selected more than 1200 Fab clones, of which 475 clones showed positive reaction to SFTS virions. However, all 475 clones were only reactive to the purified N protein tested by ELISA and IFA. To select human antibodies to SFTSV glycoproteins, we used purified N protein as blockage in another process of phage panning. However, no targeted Fab antibodies against glycoproteins were identified over screened additional 600 Fab clones. In addition to these human recombinant monoclonal antibodies, through screening 1000 mouse MAbs clones derived from immunization with SFTSV virions, 462 clones were identified to react with N protein, while only 16 clones were reactive to glycoprotein Gn or Gc expressed in recombinant baculovirus transfected SF9 cells (Figure S1).

**Figure 1:**
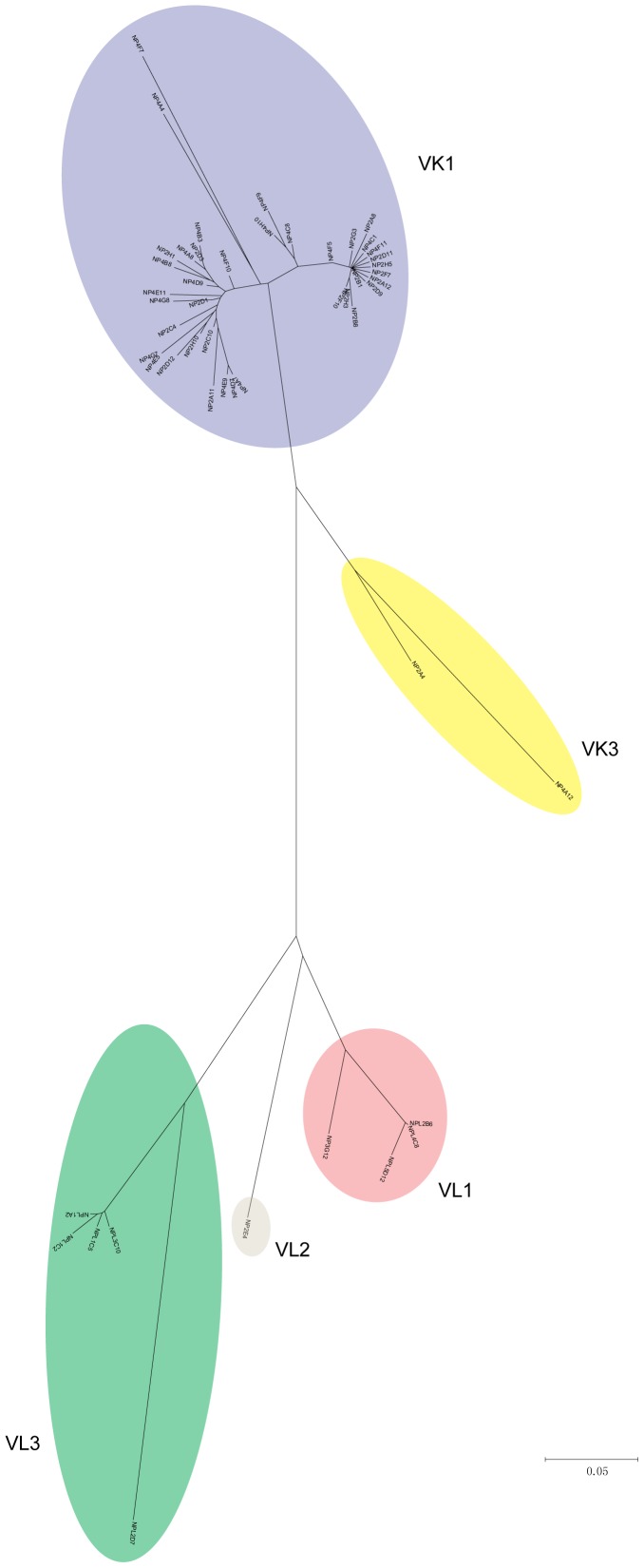
Neighbor-joining phylogenetic tree of the light chains genes of Fab antibodies. The phylogenetic tree was based on amino acid sequence in the variable regions of light chains. Among the 94 human Fab clones, 82 clones in purple represent human germline genes as specified by the VK1 family, 2 belong to VK3 family, 4 belong to VL1 family, 1 belongs to VL2 family, and 5 belong to VL3 family.

Sequence analysis of all 475 human positive Fab clones revealed the presence of 94 unique ones. Using the DNAPLOT program for alignment to the VBASE database [15], the germ line and gene families of these isolated Fab antibodies were identified. The heavy chain genes of all 94 positive clones belonged to VH3 family, while 82 of these positive clones were of VK1 family and the rest possessed distinct VK3, VL1, VL2 and VL3 families (Figure 1). As shown in Table 1, 45 unique sequences in complementarity determining regions (CDRs) of heavy chain were aligned from the 94 unique heavy and light chain combinations. Overall, CDR1 and CDR2 have less sequence variation, in contrast, CDR3 shows the greatest variability. The high frequency of V-D-J recombination and amino acid diversification found in CDR3 region might represent the process known as affinity maturation *in vivo*, suggesting that N protein was a highly immunogenic antigen in humoral immune responses to the SFTSV infection.

**Table 1:** Amino acid sequences of the complementarity determining regions (CDRs) in 45 unique heavy chains of human Fab clones isolated from phage antibody library.

Fab clones^b^	CDR1	CDR2	CDR3
H2A12^a^	SYSMN	SISSSSSYIYYADSVKG	EDYGL*****LDY
H2B10^a^	-----	-----------------	-T---*****---
H2A1	-----	-----------------	-S--P*****---
H4G1	----S	-----R-----------	-T---*****---
H2D3	-----	----G------------	-S--P*****I--
H2F8^a^	-----	----GGN----------	-S--P*****---
H4D9	-----	----R------------	-T--P*****---
H4B7	-----	----G------------	-L--N*****---
H4A4^a^	-----	----G------------	DS-H-*****F--
H2B8^a^	-----	----G------------	DL--P*****---
H2C11^a^	-----	----G------------	DS--V*****-G-
H4C1	-----	----G------------	DN--I*****F--
H2C2	-----	----G------------	D---V*****S-V
H2C10^a^	-----	----R------------	DN--I*****F--
H4A6	T----	----R------------	DN-A-*****M-V
H4E12	-----	----N------------	DS--P*****--V
H4E5	-----	F----------------	DT--I*****F--
H4B2	-----	-----------------	D----*****I--
H2D7	----S	-----------------	D---V*****-H-
H2F10^a^	-----	-----------------	D---S*****-SA
H2D12^a^	-----	-----------------	DN--I*****F--
H4F7^a^	-----	-----------------	DS---*****F--
H4G5	-----	-----------------	DN---*****F--
H2A8^a^	-----	-----------------	DQ--V*****I--
H4E3	-----	-----------------	DL--V*****V-I
H4A7	-----	-----------------	-N--V*****I--
H2B4	-----	-----------------	-----*****M-V
H2B1^a^	-----	-----------------	-N---*****M-V
H2A6	-----	-----------------	DS--I*****M-V
H2F5	-----	-----------------	DS--F*****M-V
H4G4	-----	-----------------	----V*****-YV
H2A4^a^	-----	-----------------	D---I*****S--
H4C9	-----	----G------------	RN--E****YS--
H4B3	-----	----G------------	TPG-IAVAEFM-V
H1C2^a^	-----	-----------------	TPG-IAVAEFM-V
H2D1	T----	-----------------	TPG-IAVAEFM-V
H4C8^a^	-----	-----------------	HFV-ASTH**T--
H2E4^a^	-----	-----------------	GHV-ATD***---
H2F4^a^	T----	-----------------	D---VIR***F-P
H4E11^a^	-S---	----GG-----------	GGEYSSG***F--
H5D12	-----	----G------------	LAA-ASSS**I--
H4B9^a^	-----	----G------------	TPG-GDYYY*M-V
H5A2	-----	-----------------	DL-YGDYV**---
H2H9^a^	-----	-----------------	VT-YGSGSYFF--
H5D7	--W-H	R-YTDG-STS-------	GSSYYG****M-V

a MAbs labeled were included in further study; ^b^: MAbs derived from SFTS patients were designated as “H” for the first letter, and mouse derived MAbs were designated as “M” for the first letter; -: Indiated the identical to the amino acid sequence of Fab H2A12; *: No amid acid.

**Figure 2:**
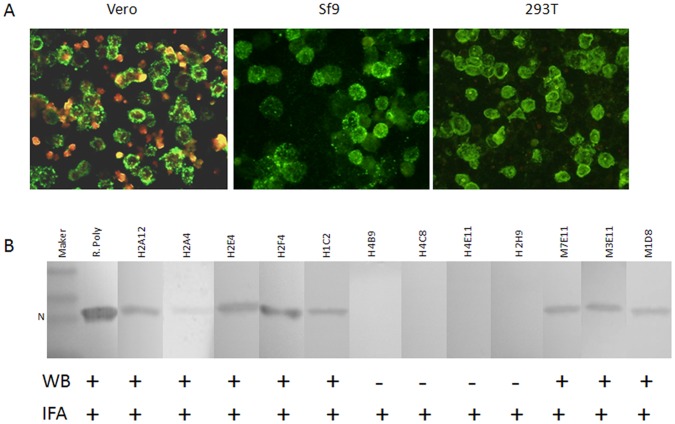
Characterization of human and mouse MAbs to SFTSV N protein. A) IFA analysis shows all selected MAbs are reactive to the viral and recombinant N protein. Vero: Vero cells infected by SFTSV strain HB29; Sf9: Sf9 cells expressing recombinant N protein with the baculovirus system; 293T: 293T cells transiently expressing the recombinant N protein. B) Western blot assay of human and mouse MAbs. Most MAbs represented a positive N protein band at 27 KDa, while MAbs H4B9, H4C8, H4E11 and H2H9 showed negative result. R. poly: rabbit polyclonal antibody immunized with the SFTSV N protein.

Next, above N protein specific Fab antibody clones with unique sequences were selected and converted into IgG molecules based on their sequence diversity and specific N protein binding activity (Figure 2A). 20 human IgG antibodies and 11 mouse MAbs to the N protein were further analyzed by western blot analysis. We found that 16 selected human MAbs and all 11 mouse MAbs were reactive to the denatured N protein of SFTSV, and showed a positive protein band of 27 KDa in western blot assay as presented in figure 2B with 5 human antibodies (H2A12, H2A4, H2E4, H2F4 and H1C2) and 3 mouse MAbs (M7E11, M3E11 and M1D8), while 4 human MAbs including H2H9, H4B9, H4C8 and H4E11 did not appear the positive binding. The different binding properties of above human and mouse MAbs in western blot assay indicated the antibodies recognized either linear epitope or conformational epitope in the SFTSV N protein. However, none of the N protein specific antibodies had the neutralizing activities. In contrast, 9 of 16 mouse MAbs targeted to SFTSV glycoprotein Gn or Gc showed significant neutralizing activities at various levels, and none of them reacted with denatured viral antigen in western blot assay (Table S1).

### Competitive ELISA Assay of Monoclonal Antibodies Against N Protein

In order to characterize epitope binding patterns of the 20 human MAbs and 11 mouse MAbs with SFTSV N protein, a set of competitive ELISA experiments were performed. Based on above analysis data, we chose three human MAb H2A12, H2H9,H3E11 and one mouse MAb M1D8 as detective antibody labeled with horseradish peroxidase (HRP), and totally 20 human MAbs and 11 mouse MAbs were used as competing antibodies in the test. As shown in Table 2, except H2H9, all human MAbs showed obvious competitive binding activity to N protein with H2A12, including human MAbs H4B9, H4C8 and H4E11, which were potential conformational epitope binder as shown in the WB assay. Further, competition of mouse MAbs with H2A12 also showed that 9 of the 11 mouse MAbs, represented by M7E11, obviously blocked the binding activity with human MAbs H2A12. However, 2 mouse MAbs M3E11 and M1D8 did not block the binding of H2A12, indicating that the N protein epitopes recognized by the two mouse MAbs were probably different from the epitope recognized by human MAb H2A12. Furthermore, we selected above 3 MAbs H2H9, M3E11 and M1D8 which were non-competitive with H2A12 as detective antibodies to determine the binding competition of each of the 3 MAbs with all human and mouse MAbs. As showed in Table 2, none of the 3 MAbs showed competition with other MAbs. Thus, there were at least 4 distinct binding sites in N protein, represented by the MAbs of H2A12, M3E11, M1D8 and H2H9, and the epitope of MAbs H2A12 was the major binding site of N protein for most human and mouse MAbs.

**Table 2:** Competitive ELISA assay of monoclonal antibodies against N protein.

MAbs^a^	HRP conjugated MAb (%)^b^			
	H2A12	H2H9	H3E11	M1D8
H2A12	93	22	25	12
H2A4	75	16	17	21
H2E4	80	22	27	31
H2F4	77	19	22	25
H1C2	78	24	23	14
H4B9	80	22	31	11
H4C8	82	27	17	12
H4E11	80	31	13	11
H2H9	32	88	19	12
M7E11	88	30	15	32
M3E11	20	15	85	19
M1D8	20	23	14	90

a Competing MAbs, SFTS patients derived were designated as “H” for the first letter; and mouse derived were designated as “M” for the first letter.

b Percentage of binding inhibition of HRP conjugated detective antibodies in competitive ELISA assay.

### Computer Modeling and Docking of Interaction between Antibody and N Protein

**Figure 3:**
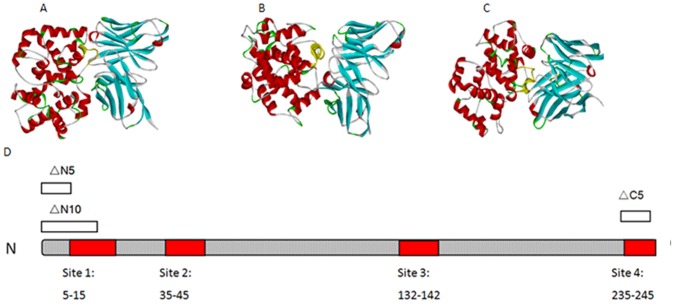
Design for single mutations of SFTSV-N protein. Computer-simulated docking structure shows 3 representative binding sites on N protein at A) amino acid 8–15, B) amino acid 35–45, and C) amino acid 132–142. D) The scheme represents the regions of site-directed mutations in N protein based on 3D-structure predictions and truncation studies.

Since the epitope recognized by a panel of MAbs represented by H2A12 might be a dominant epitope of SFTSV N protein, we further define potential epitopes on N protein surface recognized by H2A12. We first found an antibody crystal structure 1DFB (PDB) which has as high as 90% sequence similarity with human MAbs H2A12, and use this structure as the model in computer-simulation. The crystal structure of N protein of the rift valley fever virus (RVFV) was selected as model for SFTSV N protein, because RVFV N protein has maximal identity of 41.4% in backbone structures with SFTSV N protein [1], and more importantly, they both belong to the genus *Phlebovirus* in the *Bunyaviridae* family with similar structure [16]. After using Energy minimization and molecular dynamic optimization to optimize the structure, we used DOCK program for geometric docking simulation to mimic the possible interaction of the antibody and N protein models. Computer-simulation predicted fragments of amino acids 8–15, 35–45, 74–89, 113–116, 132–142, and 217–222 in N protein, which had highest scores as key contact sites by the RDOCK program, as possible binding sites of antibody (Figure 3). The amino acids selected by computer-simulation were further confirmed using site-directed mutagenesis *in vitro* to validate the predicted epitopes in real protein-protein interaction between SFTSV N protein and human MAbs.

### Epitope Mapping by Site-directed Mutations

Since previous studies on *Hantavirus*, RVFV and *Uukuniemi* virus (UUKV) showed that terminal regions of viral N protein are especially important for its function [16], [17], [18], we tested the both N and C terminal truncated protein first. We found that the N protein with truncation of 5 amino acid at N terminus (ΔN5) was recognized by all tested antibodies listed in Table 3, while the antibody binding activity of the N protein with truncation of 10 amino acid at N terminus (ΔN10 ) or 5 amino acid at C terminus (ΔC5) was significantly declined in IFA tested with the N protein specific rabbit polyclonal antibody (Table 3), and even abolished tested with those human or mouse MAbs. However, the binding activity of ΔN10 or ΔC5 with rabbit polyclonal antibodies was remained in western-blot assay (Figure S2).

**Table 3:** Epitope mapping of MAbs to a panel of site mutations and truncations of SFTSV N protein tested by IFA assay.

Antibodies	WT N	ΔN5	ΔN10	ΔC5	S9A^a^	E10A^a^	E10A F11A^a^	F11A^a^	I39A^b^	K40A^b^	K41A^b^	E44A^b^	T45A^b^	N139A^c^	Y140A^c^	M141A^c^	Y242A^d^	R243A^d^	N244A^d^
R. Poly	+++	+++	+	+	+++	+++	+++	+++	+++	+++	+++	+++	+++	+++	++	+++	+++	+++	+++
H2A12^e^	+++	+++	–	–	+++	+	–	+++	+++	+++	+++	+++	+++	+++	+	+++	+++	+++	+++
H2A4^e^	+++	+++	–	–	+++	–	–	+++	+++	+++	+++	+++	+++	+++	+	+++	+++	+++	+++
H2E4^e^	+++	+++	–	–	+++	+	–	+++	+++	+++	+++	+++	+++	+++	+	+++	+++	+++	+++
H2F4^e^	+++	+++	–	–	+++	–	–	+++	+++	+++	+++	+++	+++	+++	+	+++	+++	+++	+++
H4B9^e^	+++	+++	–	–	+++	++	–	+++	+++	+++	+++	+++	+++	+++	+	+++	+++	+++	+++
H4C8^e^	+++	+++	–	–	+++	–	–	+++	+++	+++	+++	+++	+++	+++	+	+++	+++	+++	+++
H4E11^e^	+++	+++	–	–	+++	+	–	+++	+++	+++	+++	+++	+++	+++	+	+++	+++	+++	+++
H2H9	++	++	–	–	++	++	++	++	++	++	++	++	++	++	+	++	++	++	++
H1C2	+++	+++	–	–	+++	+++	+++	+++	+++	+++	+++	+++	+++	+++	+	+++	+++	+++	+++
M7E11^e^	+++	+++	–	–	+++	+	–	+++	+++	+++	+++	+++	+++	+++	+	+++	+++	+++	+++
M1D8	+++	+++	–	–	+++	+++	+++	+++	+++	–	–	–	+++	+++	+	+++	+++	+++	+++
M3E11	+++	+++	–	–	+++	+++	+++	+++	+++	+++	+++	+++	+++	+++	++	+++	+++	–	+++

a–d Single alanine substitution at amino acid positions ^a^5∼15 of N-terminal; ^b^positions 35∼45; ^c^positions 132∼142, ^d^positions 235∼245 of C-terminal. ^e^Refer to major MAbs representing abolished or significantly reduced binding activity to mutation of E10A in N protein; (+) to (+++) indicates the relative intensity of fluorescence. R. Poly: a rabbit polyclonal antibody immunized with the SFTSV recombinant N protein.

**Figure 4:**
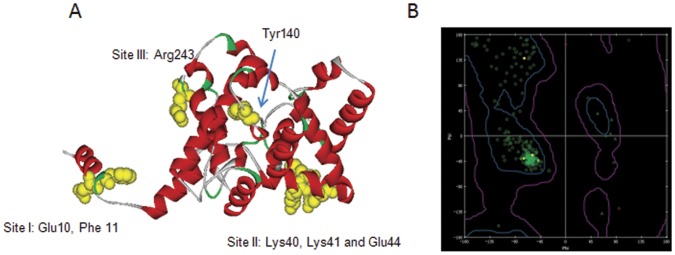
Epitope mapping of SFTSV N protein by 3D structure prediction. Critical amino acids for antibody binding are indicated in the modelling structure as the sites they located (Yellow). B) Ramachandran plot of the N protein prediction illustrates that visualized backbone dihedral angles (phi/psi) of amino acid residues in modeling structure are properly validate.

In order to address which amino acid sites were critical for MAbs binding and the antigenic epitopes of N protein, single mutations with alanine substitution were performed. For N terminus, single alanine mutations of N terminal 5∼15 amino acid resulted in no change of antibody binding activities, but only the mutation of Glu10 to Ala (E10A) significantly reduced the binding activity of most human MAbs represented by H2A12, and most mouse MAbs represented by M7E11 (Table 3). The mutation of E10A combining with mutation of Phe11 to Ala (F11A) resulted in the complete loss of binding with those MAbs, while single mutation of F11A had no obvious reduction in binding activity to above MAbs. Mutation analysis of C-terminal fragment 235∼245 showed that only mouse MAbs M3E11 showed no binding activity to the single mutation of Arg243 to Ala (R243A), while the binding activity of rest antibodies was not affected. Further, based on predicted antibody binding sites from computer modeling, we did single mutations on all 6 amino acids fragments identified. The results showed that, at amino acid positions 38∼45, substitution of Lys40 to Ala (K40A), Lys41 to Ala (K41A) and Glu44 to Ala (E44A) were indispensible to the binding of mouse MAbs M1D8, while not affected binding with all other MAbs (Table 3). The mutation analysis of amino acid 132∼142 showed that mutation of Tyr140 to Ala (Y140A) generally reduced the binding activity of all tested antibodies including polyclonal antibody. Alanine mutations in amino acid 74–89, 113–116 and 217–222 showed no reduction in the binding activity with all MAbs. Therefore, the critical amino acids in epitopes of the N protein for antibody binding are listed in the modeling structure (Figure 4A), and Ramachandran plot of the SFTSV N protein model illustrated that visualized backbone dihedral angles of amino acid residues were properly validated (Figure 4B). However, all above single mutations did not reduce any binding activities of human antibodies H1C2 and H2H9, and the latter one was suspected to have an independent binding site different from others according to the competition ELISA assay (Table 2).

## Discussion

In this study, we for the first time reported the generation and characterization of a panel of human MAbs by using phage display technology. The critical role of N-terminal amino acids for the immunogenicity of SFTSV N protein as well as other 3 antigenic sites were finally defined.

As a newly emerged viral pathogen, successful control of SFTSV epidemic requires a better understanding of antigen targets in humoral immune responses to the infection. Results in this paper presented that intensive antibodies were reactive to the N protein, but less antibodies to viral glycoprotein, which is believed to be major neutralizing antibody inducer. In general, all virus infection should induce neutralizing antibodies, as for bunyavirus infection, it has been largely evidenced that almost all of neutralizing polyclonal or monoclonal antibodies are viral glycoprotein Gn or Gc specific [19], [20], but not for N protein specific antibodies, even the antibodies to some strong immunogenic N protein epitopes had no neutralizing activity [5], [21]. The large numbers of human antibodies derived from SFTS patient antibody phage display library finally turn out to the N protein, it is possible that the abundant amount and high immunogenicity of the N protein produced large amount of antibodies with high binding affinity to N protein distract the selection of neutralizing antibodies to glycoprotein in the phage panning process, as for other *Bunyavidridae* such as RVFV and *Puumala* virus, it has been reported that the reaction between antibody and glycoprotein involves conformational epitopes that are more fragile [5], [22], [23]. In addition, the SFTS virions we used for antibody library screening might not be sufficiently native in the process of phage panning. The undergoing effort for purifying native glycoprotein would facilitate identifying MAbs against glycoprotein.

However, the large number of specific antibodies against SFTSV N protein gained in this study also indicated that the N protein played an important role in humoral immune responses to the new bunyavirus infection and the process of affinity maturation *in vivo* after infection aggravated the inducing of numerous antibodies. These data are coincident with previous reports from other members in *Phlebovirus* genus, family *Bunyaviridae.* For members in *Phlebovirus* genus, the N protein is the most abundant viral product in virions and virus infected cells [3]. It is reported that the N protein of RVFV was reported to be the immunodominant viral protein, and antibodies against N were readily detected early after infection and in convalescent individuals, providing robust basis for diagnostic detection of the disease [4], [24], [25]. Also, the nucleoprotein of Toscana virus was identified as the major antigen responsible for both IgM and IgG in Toscana virus infected patients by immunoblotting and semiquantitative radioimmunoprecipitation assays [5]. Although it is generally agreed that the N proteins of the bunyaviruses dose not induce neutralizing antibodies, even antibodies to the strongly immunogenic N protein of RVFV had no neutralizing activity [26], [27], protection studies conducted in vivo demonstrated that monoclonal antibodies to the N protein partially protect animals from challenge with the virulent virus [5], [21]. Similar results have been obtained in *Hantavirus* infections where antibodies to the N protein have been shown to protect mice but no neutralizing activities in vitro [28], [29]. We also tested more than 20 human MAbs and none of them had neutralizing function. Although the exact role of various MAbs induced by the SFTSV N protein is not clear, at least, we consider that the N protein induced intense specific antibody responses, and served as an important antigenic target for diagnosis of SFTS.

As an emerging infectious disease, epitopes in the N protein of SFTSV has not been identified. In fact, there were few reports on epitopes of viral N proteins even for other members in the *Phlebovirus* genus of *Bunyaviridae* family. It was reported that at least three different epitopes of RVFV N protein were defined through competition ELISA assay, and the amino acid residue 159 might be an important residue for antibody binding [4]. In this study, we found that the amino acids at positions N10, N11 of N-terminus and C243 of C-terminus were significantly needed for keeping the immunogenicity of SFTSV N protein. In fact, the N- and C- terminus are reported to be important for oligomerization and stabilization of N protein of *Phlebovirus*. The oligomerization of N monomers is an essential requirement for RNA packing in mature particles. The polymer of N protein can provide adequate templates for RNA binding, and contribute to the formation of inclusion body in the cytoplasm of virus infected cells [17], [30]. The oligomerization of UUKV N protein depends on the presence of intact helices on both termini of the N protein molecule and that a specific structure in N-terminal region plays a crucial role in the N-N interaction [18]. The N terminal amino acids 1 to 70 of RVFV are also involved in the dimmer formation as reported [17]. The crystallized RVFV N protein validated that N protein has a compact helical fold consisting of N-terminal and C-terminal lobes of approximately equal size, and the N terminal residues (Tyr4, Phe11) make intra- and inter- molecular contacts with hydrophobic residues to stabilize the hydrophobic core of the N protein [16]. Therefore, the immunogenicity of N- and C- terminus of SFTSV N protein might associate with their functions to impact the oligomerization and stabilization of N protein.

In addition, according to the results of competition assay, at least four distinct antigenic sites were recognized by MAbs, represented by human MAbs H2A12, H2H9, and mouse M3E11, M1D8. Mutation analysis revealed critical amino acid residues for binding with MAbs H2A12 group, M1D8, M3E11, however, the binding sites of H2H9 still remain unclear. Although interface of antigen and antibody is best defined by crystallography, computer-simulated modeling combined with site-directed mutagenesis has been used successfully to define the sites critical for the interaction between antibody and antigen [31]. We also performed the synthesized peptide mapping which is commonly used for the epitope mapping study, but our results were not productive probably due to the inherent complexity of the viral antigen (Data not shown). Using specific MAbs to analyze viral evolutions with natural gene selection will give us more information on critical epitope mapping of SFTS N protein, and this certainly requires more investigation.

In summary, we generated and characterized a panel of human MAbs against nucleocapsid protein of SFTS virus derived from SFTSV infected patients using phage display technology. Importantly, localization of at least 4 antigenic sites on nucleocapsid protein recognized by a panel of human MAbs has provided, for the first time, insight into the human antibody responses to SFTS viruses which contribute to the SFTS immunity in the recovered patient. Furthermore, the critical role of N- terminal amino acids for the immunogenicity of SFTSV N protein would help to understand the significance of the defined epitopes and applications for diagnosis of human infection with SFTS virus.

## Supporting Information

Figure S1 Characterization of mouse MAbs to glycoprotein Gn and Gc by IFA. The antigen slides were made from Vero cells infected with SFTSV strain HB29, or Sf9 cells expressing Gn or Gc protein with infection of recombinant baculovirus. Mouse MAbs represented by 2D5 showed Gn specificity on both Vero and Sf9 cells (Vero-Gn, Sf9-Gn); mouse MAbs represented by M1G8 showed Gc specificity on both Vero and Sf9 cells (Vero-Gc, Sf9-Gc).
(TIF)

Figure S2 IFA and western blot assay of truncated N protein from the 293T lysate with rabbit polyclonal antibody. The intact N and truncated N proteins were transiently expressed in 293T cells and the cells tested by IFA (A) with R. poly antibodies showed a positive reactivity to the intact N and truncated △N5, and a weak intensity of fluorescence to truncated △N10 and △C5 proteins. The cell lysates were used for western blot assay of above truncated N protein and showed all positive reactivity (B). R. poly: A rabbit polyclonal antibody immunized with the SFTSV N protein; N: The intact N protein transiently expressed in293T cells; △N5, △N10 and △C5: Truncation of 5, 10 amino acids at the N- or C-terminus of N protein; NC: Normal 293T cells as negative control.
(TIF)

Table S1 Neutralization activity of human and mouse MAbs against SFTSV tested by micro-neutralization test.
(DOC)
