# Supplementary figures and images for: Critical Epitopes in the Nucleocapsid Protein of SFTS Virus Recognized by a Panel of SFTS Patients Derived Human Monoclonal Antibodies
Source: PLoS One. 2012 Jun 12;7(6):e38291. doi: 10.1371/journal.pone.0038291 (PMC3373585; doi:10.1371/journal.pone.0038291)

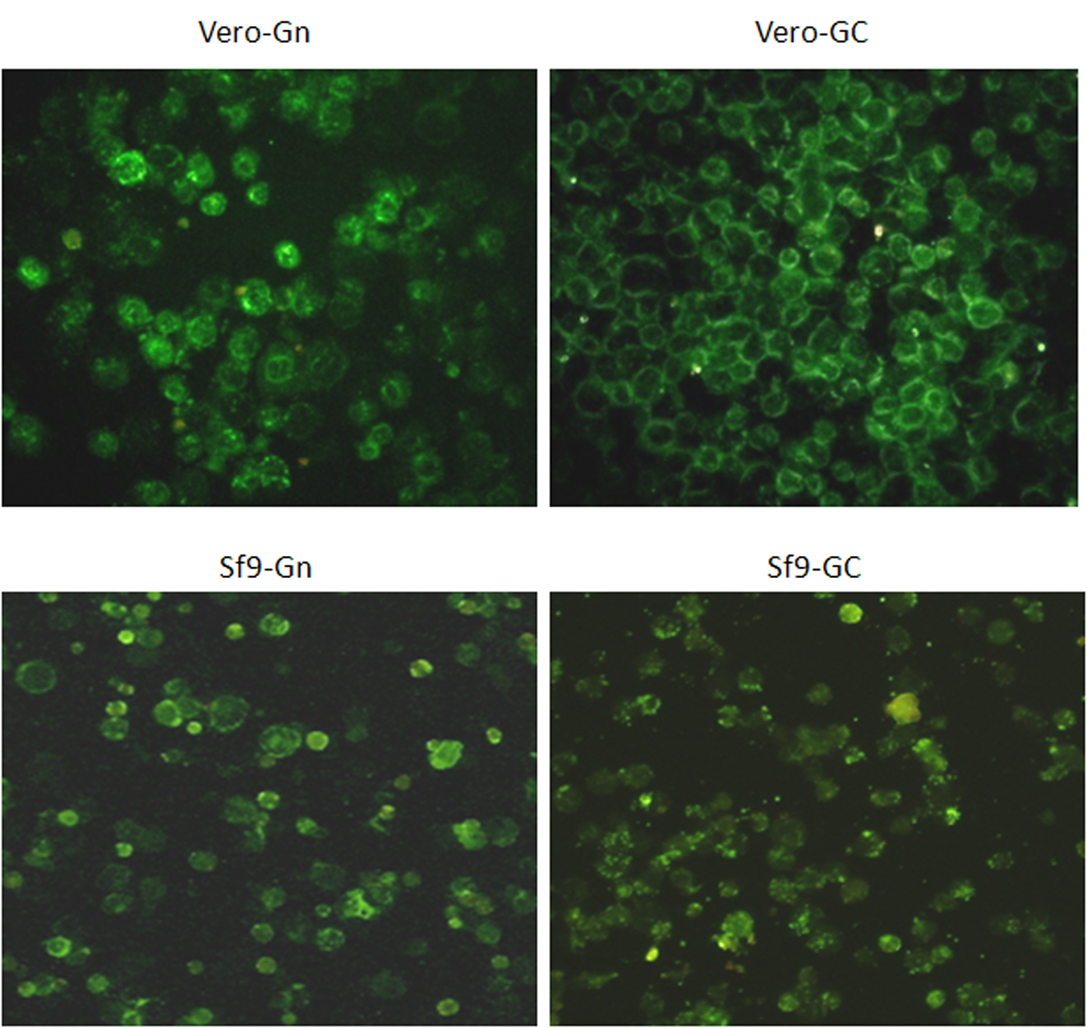

Supplement: Figure S1 — Characterization of mouse MAbs to glycoprotein Gn and Gc by IFA. The antigen slides were made from Vero cells infected with SFTSV strain HB29, or Sf9 cells expressing Gn or Gc protein with infection of recombinant baculovirus. Mouse MAbs represented by 2D5 showed Gn specificity on both Vero and Sf9 cells (Vero-Gn, Sf9-Gn); mouse MAbs represented by M1G8 showed Gc specificity on both Vero and Sf9 cells (Vero-Gc, Sf9-Gc). (TIF) [file pone.0038291.s001.tif]

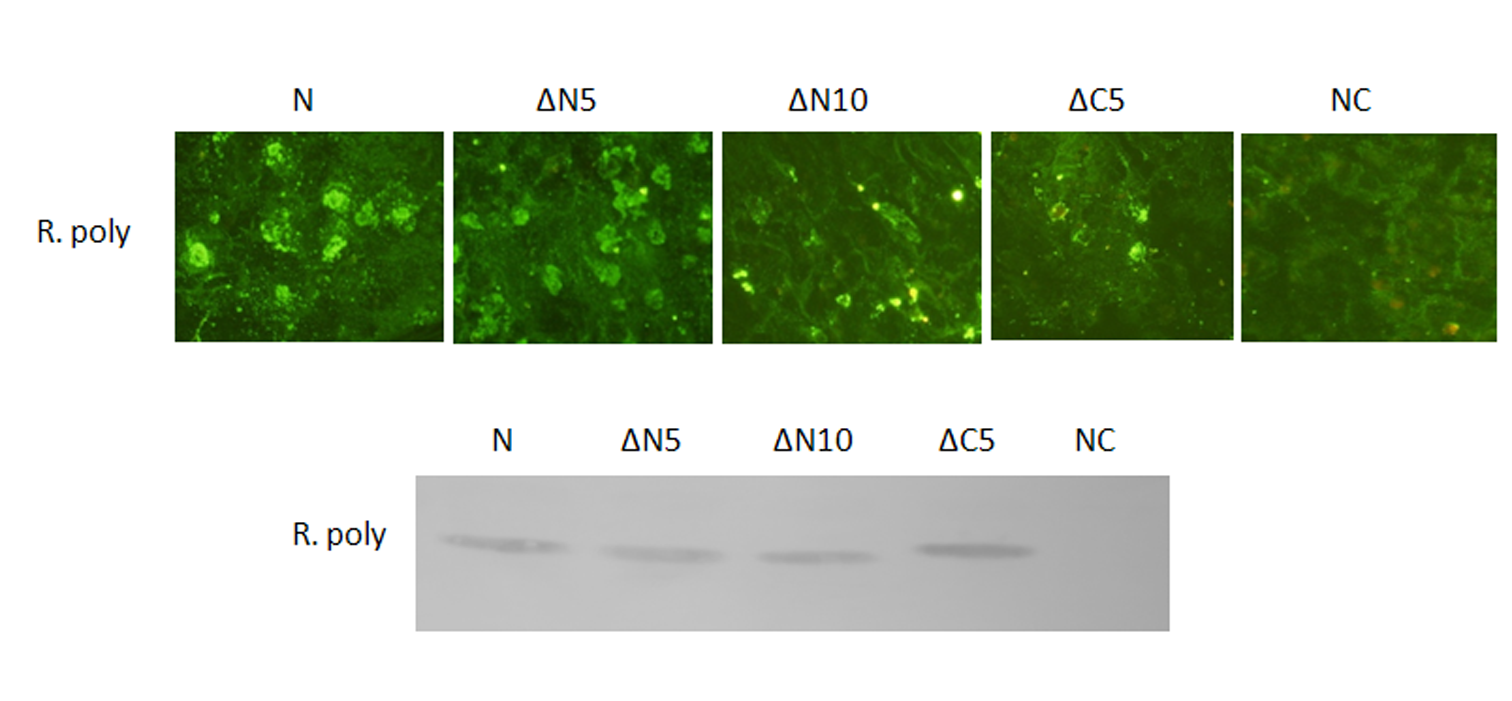

Supplement: Figure S2 — IFA and western blot assay of truncated N protein from the 293T lysate with rabbit polyclonal antibody. The intact N and truncated N proteins were transiently expressed in 293T cells and the cells tested by IFA (A) with R. poly antibodies showed a positive reactivity to the intact N and truncated △N5, and a weak intensity of fluorescence to truncated △N10 and △C5 proteins. The cell lysates were used for western blot assay of above truncated N protein and showed all positive reactivity (B). R. poly: A rabbit polyclonal antibody immunized with the SFTSV N protein; N: The intact N protein transiently expressed in293T cells; △N5, △N10 and △C5: Truncation of 5, 10 amino acids at the N- or C-terminus of N protein; NC: Normal 293T cells as negative control. (TIF) [file pone.0038291.s002.tif]
