# Supplementary material for: Critical Epitopes in the Nucleocapsid Protein of SFTS Virus Recognized by a Panel of SFTS Patients Derived Human Monoclonal Antibodies
Source: PLoS One. 2012 Jun 12;7(6):e38291. doi: 10.1371/journal.pone.0038291 (PMC3373585; doi:10.1371/journal.pone.0038291)
Supplement: Table S1 — Neutralization activity of human and mouse MAbs against SFTSV tested by micro-neutralization test. (DOC) [file pone.0038291.s003.doc]

**Table S1.** Neutralization activity of human and mouse MAbs against SFTSV tested by microneutralization test.

| **MAbs** | | **Targeted antigen** | | **MNT^c^** |
| --- | --- | --- | --- | --- |
|  |  | **IFA^a^** | **WB^b^** |  |
| Human | H2A12 | N | N | ＜10 |
|  | H2A4 | N | N | ＜10 |
|  | H2E4 | N | N | ＜10 |
|  | H2F4 | N | N | ＜10 |
|  | H1C2 | N | N | ＜10 |
|  | H4B9 | N | - | ＜10 |
|  | H4C8 | N | - | ＜10 |
|  | H4E11 | N | - | ＜10 |
|  | H2H9 | N | - | ＜10 |
| Mouse | M7E11 | N | N | ＜10 |
|  | M3E11 | N | N | ＜10 |
|  | M1D8 | N | N | ＜10 |
|  | M2D5 | Gn | - | 1280 |
|  | M1C8 | Gn | - | 80 |
|  | M6B12 | Gn | - | 20 |
|  | M3B7 | Gn | - | 40 |
|  | M6G3 | Gn | - | 1280 |
|  | M6G10 | Gn | - | 20 |
|  | M1G8 | Gc | - | 160 |
|  | M4C7 | Gc | - | 40 |
|  | M7D8 | Gc | - | 20 |

^a^ Immunofluorescence assay (IFA) was performed with SF9 cells expressing the nucleocapsid protein (N) or glycoprotein (Gn or Gc).

^b^ Western blot assay (WB) was performed with purified SFTSV virions. The appearance of positive band of N protein is indicated by “N”, and negative result without positive bands is indicated by “-”.

^C^ Microneutralization test (MNT) was performed with serially diluted mouse hybridoma ascites or purified human recombinant antibodies (1mg/ml). The end-point titer was expressed as the reciprocal of the highest dilution factor that prevented infection.
